# First results of MIRABEL: a research program to develop tools for risk and cost/benefit analysis of peanut allergy

**DOI:** 10.1186/2045-7022-3-S3-P135

**Published:** 2013-07-25

**Authors:** A Crépet, CF Elegbede, S Marette, D-A Moneret-vautrin, A Papadopoulos, J Roosen

**Affiliations:** 1 DER, ANSES, Maisons-Alfort, France; 2 Public Economy, INRA, Thiverval-Grignon, France; 3 Allergo-Vigilance Network, Vandoeuvre Les Nancy, France; 4 TUM, München, Germany

## Background

Traces of allergenic food represent a significant health risk for persons who have an allergy to specific food. Knowledge is missing on the different risk components and particularly on the link between presence of traces of allergens in food, food consumption behaviours of allergic sufferers and thresholds of reaction. Moreover, there is a lack of risk-based guidance and operational tools of those involved in allergen risk management, who are food industries, the allergic individuals and the food regulators. Peanut is associated with the highest prevalence of food allergy and is one of the food allergens that lead to the most severe adverse effects.

## Methods

MIRABEL program involves ANSES, INRA and AllergyVigilance Network in order to deal with medical research, dietary survey, chemical analysis, socio-economics and applied mathematics. Each risk component will be investigated to accurately characterize the risk of peanut allergy and to test different risk management options. Accurate data will be collected on clinical characteristics of peanut allergic patients, thresholds of reactions, consumptions and behaviours relating to peanut-containing products (chocolate, cereals or biscuits, etc). Peanut presence will be analyzed in the most consumed products. Methodological developments in Bayesian statistics and probabilistic modelling will be realized to be able to combine the acquired data in an integrated risk quantification model. A cost-benefits analysis for the stakeholders (food industry, allergic individuals and regulators) will also be conducted to anticipate impacts of new policies.

## Results

The patient survey started in April 2012 with 83 allergists in town offices and hospitals. To date, 450 medical questionnaires were filled by 40 allergists. For 120 patients among 148 tested, oral diagnostics gave positive results. The rate of complete dietary questionnaires filled by the patients is of 50% for the paper and of 30% for the online questionnaires. The age of the patients ranges from 2 to 39 years with a median of 9 years. From the first results, the sampling plan for monitoring allergens in consumed food by allergic sufferers was designed. It was observed that 17% of consumed products were labelled as “may contain peanut traces”. The products collection will be conducted in January 2013.

## Conclusion

The program aims to set an integrated and operational framework for the allergic risk analysis, in order to improve quality of life of allergic sufferers.

## Disclosure of interest

None declared.

